# Extracellular Vesicles in *Flaviviridae* Pathogenesis: Their Roles in Viral Transmission, Immune Evasion, and Inflammation

**DOI:** 10.3390/ijms25042144

**Published:** 2024-02-10

**Authors:** Anastasia Latanova, Vadim Karpov, Elizaveta Starodubova

**Affiliations:** Engelhardt Institute of Molecular Biology, Russian Academy of Sciences, 119991 Moscow, Russia; karpov@eimb.ru (V.K.); estarodubova@yandex.ru (E.S.)

**Keywords:** extracellular vesicles, exosome, microvesicle, *Flaviviridae*, pathogenesis, inflammation, immune evasion

## Abstract

The members of the *Flaviviridae* family are becoming an emerging threat for public health, causing an increasing number of infections each year and requiring effective treatment. The consequences of these infections can be severe and include liver inflammation with subsequent carcinogenesis, endothelial damage with hemorrhage, neuroinflammation, and, in some cases, death. The mechanisms of *Flaviviridae* pathogenesis are being actively investigated, but there are still many gaps in their understanding. Extracellular vesicles may play important roles in these mechanisms, and, therefore, this topic deserves detailed research. Recent data have revealed the involvement of extracellular vesicles in steps of *Flaviviridae* pathogenesis such as transmission, immune evasion, and inflammation, which is critical for disease establishment. This review covers recent papers on the roles of extracellular vesicles in the pathogenesis of *Flaviviridae* and includes examples of clinical applications of the accumulated data.

## 1. Introduction

*Flaiviviridae* include several human viruses transmitted either through blood-to-blood contact and sexual intercourse, such as the hepatitis C virus (HCV), or by arthropods, such as the Zika virus (ZIKV), the dengue virus (DENV), the West Nile virus (WNV), the tick-borne encephalitis virus (TBEV), the Japanese encephalitis virus (JEV), and the yellow fever virus (YFV), causing acute and chronic diseases with mild-to-severe symptoms, including liver inflammation, neuroinflammation, endothelial damage, and even death. DENV and HCV account for the largest number of *Flaviviridae* infection cases worldwide, with an estimated 390 mln infections per year and 96 mln symptomatic cases for DENV [1] and 56.8 mln HCV-infected individuals [2]. The burden of *Flaviviridae* infections may increase in the coming years due to global warming and subsequent changes in insect habitat, accessibility of human movement, and other reasons. At the same time, prophylactic and therapeutic options for the prevention and treatment of *Flaviviridae* are limited. Approved prophylactic vaccines include only those against DENV [3], JEV [4], YFV [5], TBEV [6], and, in the case of DENV, the vaccine formulation is suboptimal, with variable efficacy against four DENV serotypes and the risk of antibody-dependent enhancement of infection [7]. In terms of therapeutic drugs, only antivirals against HCV are currently approved for clinical use [8], while clinical trials are underway for the other flaviviruses. A better understanding of *Flaviviridae* pathogenesis is urgently needed to accelerate trials of prophylactic and therapeutic options.

In general, viral pathogenesis involves several steps preceding the onset of viral disease. These steps include the following: virus entry, primary viral replication, virus spread within the host, infection of cells with special affinities for the virus, cellular injury, host immune response, viral clearance or persistence, and viral shedding and transmission [9]. During these steps, a virus utilizes host resources and interacts with host factors. Among these steps, virus transmission and spread, evasion of the host immune response, and tissue-specific inflammation, caused by infection and cellular injury, appear to be of great importance for flaviviral pathogenesis.

*Flaviviridae* pathogenesis may be affected by host extracellular vesicles, as shown by research over the last decade. Of note, early studies of extracellular vesicles (EVs) considered them to be cell culture artifacts or the product of specialized cell types, but, later, their recognition as universal transmitters of signaling molecules changed the understanding of cellular communication [10]. Viral infections have been shown to modulate the amount and content of EVs. Thus, research on EVs associated with *Flaviviridae* infections may fill in the gaps in our knowledge regarding the mechanisms of viral pathogenesis, which is necessary for the development of efficient virus-targeted therapies.

In this review, we discuss how EVs influence the pathogenetic features of *Flaviviridae*, namely, transmission, immune evasion, and inflammation. The clinical aspects of the use of EVs for the development of antiviral therapeutic options are also evaluated.

## 2. Biology of EVs

EVs are membrane-bound particles released into the extracellular space by living or dead/dying cells. They are detected in all body fluids and can be isolated from the conditioned medium of cultured cells [11]. EVs are considered to be vehicles for active molecules, biomarkers, and therapeutic agents. The list of their functions can be extended to include the induction of signaling, trophic support, and clearance of cellular material. Several cellular mechanisms are involved in the regulation of the biogenesis of EVs, including the ESCRT machinery (in particular, TSG101 and ALIX proteins, known markers of EVs), tetraspanins, sphingomyelinase, etc. [12,13].

### 2.1. EV Classification and Functions

The International Society for Extracellular Vesicles (ISEV) regularly publishes *Minimal information for studies of extracellular vesicles* (*MISEV*) [14]. *MISEV* recommends the use of the generic term “extracellular vesicles” unless the subcellular origin of the EVs can be demonstrated [14,15]. It is important to note that it is extremely difficult to assign EVs to a specific biogenesis pathway, as this can only be documented by live imaging techniques immediately during EV release. Testing for EV-specific markers could help, but there is no consensus on their specificity for EV biogenesis pathways. To somehow classify EVs, *MISEV* suggests specifying their diameter (“small EVs” < 200 nm and medium/large EVs” > 200 nm) or density ranges, expression of biochemical markers, or description of EV isolation conditions/cell origin [14]. However, many researchers still use the classification system based on EVs’ biogenesis pathway, which distinguishes three main subpopulations of EVs [16,17]:

- Apoptotic bodies, which are large vesicles formed by cellular fragmentation and blebbing of the cell plasma membrane during apoptosis [18,19]. They are typically 1–5 µm in diameter.
- Ectosomes, which comprise microvesicles and some other variants of EVs such as oncosomes. Their characteristic feature is that they are formed in the plasma membrane directly from outward budding, and their size typically ranges from 100 nm up to 1 µm in diameter, more commonly >200 nm [20]. They must be centrifuged at 10,000–15,000× *g* for sedimentation.
- Exosomes, generated in the cell during the endocytic pathway due to inward budding of the endosomal membrane, with a typical size of 30–150 nm in diameter, thus requiring high-speed centrifugation (100,000× *g*) for sedimentation [16,21]. The process of exosome generation is as follows [22]: after invagination of the plasma membrane, some extracellular components and cell membrane proteins are wrapped together to form early endosomes. These early endosomes can exchange substances with other organelles or fuse to form late endosomes and intracellular multivesicular bodies (MVB), which contain numerous intraluminal vesicles (ILV). MVBs can be degraded by autophagosome/lysosome pathways or fuse with the plasma membrane to release endogenous substances and also ILVs, which, at this stage, are regarded as exosomes [22].

In addition to these subtypes, other subpopulations of EVs have been described, including migrasomes, shed by migrating cells [23], and oncosomes, shed by amoeboid cancer cells [24], among others [25,26].

The final fate of EVs can be either their direct release into biological fluids, promoting the clearance of unwanted cellular contents, or intracellular communication, when they reach both nearby cells and distant organs through circulation, thus performing regulatory functions [27]. Modes of EV communication include signaling, when EVs membrane-associated surface molecules or surface-attached nucleic acids and proteins interact with neighboring cells in a classic ligand–receptor manner [28]. The second mode is information transfer, when EVs carrying a functional cargo fuse with a recipient cell. This requires the fusion of EVs with a target plasma membrane or their internalization and fusion with an intracellular membrane, i.e., endosomal [28].

### 2.2. Current Challenges in EV Studies

The are several fundamental challenges to studies on EVs. They are not the subject of this review and are covered in detail in several recent reviews [26,27,29,30], but we would like to highlight the most important of them. First, most isolation techniques do not guarantee the purity of EV fractions. Contaminants are either derived from the isolation procedure (e.g., components of commercial kits) [14] or may be co-isolated with EVs due to size overlap (e.g., ribonucleoproteins, nucleosomes, lipoproteins, viral particles) [26]. Thus, functions attributed to EVs may be determined by these additional components or their combination with EVs. To reduce contamination in EV preparations, sequential use of different separation techniques may be recommended [29], although this inevitably complicates the experimental workflow and is time consuming. The other important issue is the heterogeneity of EV fractions. They usually contain a mixture of EV subpopulations, which makes it difficult to attribute EV functions to a specific subpopulation [31]. To solve this problem, ISEV recommends combining the high-resolution imaging of isolated EVs together with methods of measuring the size and concentration of EVs [14]. Further development of microfluidic and lab-on-chip technologies will help characterize individual EVs [30,32,33], thus solving the problem of heterogeneity and allowing the use of small sample volumes, which may be essential for biomedical and clinical applications. Another limitation of EV studies is the uncertainty about the fate of secreted EVs. In functional studies, it is difficult to determine which mode of EV communication is relevant for the described effect, signaling, or information transfer [27]. The use of selective inhibitors of specific intracellular trafficking pathways may be required to identify the cellular pathways critical for EV function. Because of all these limitations, EV studies should include detailed descriptions of the protocols used and very careful interpretations of the experimental data.

### 2.3. EVs and Viruses

It should be noted that EVs share many features with viruses, starting with their biophysical properties and similar biochemical composition [34]. Both the EV biogenesis pathway and viral replication cycles utilize the ESCRT machinery. Subsequently, like exosomes, viruses can be released via the MVB route [34]. Viruses also stimulate the production of EVs in infected cells, which carry replication-competent viruses which promote viral replication in recipient cells [35] and, thus, viral transmission not only within a host but also, as demonstrated for *Flaviviridae*, from arthropod cells to human cells [36,37,38,39,40,41,42]. By encapsulating viral components, EVs help them evade immune system recognition and may contribute to viral persistence and the establishment of chronic infection [35]. In addition to the transfer of infectious viral components, EVs from virus-infected cells transmit signals that modulate cellular processes for their benefit. On the other hand, EVs may also play a role in limiting viral infections by modulating the host immune response [35]. These roles of EVs in the pathogenesis of *Flaviviridae* will be discussed below. Finally, EVs can be used as carriers for antiviral drugs and their contents, as biomarkers of disease and its severity, so examples of clinical application of EVs in relation to *Flaviviridae* are given in the last review section.

## 3. EV Roles in the Transmission of *Flaviviridae*

Viruses can utilize EVs for their transmission in different manners. First, EVs shed from virus-infected cells may help transmit infectious viral RNA or whole virions. Of note, the flaviviral virion is approximately 40–60 nm in diameter, so, considering the diameter of EVs, not more than 6–8 virions may be packaged within EVs. Viral RNAs and proteins, therefore, allow a greater amount of infectious material to be enclosed inside EV compared to the whole virion [43]. Table 1 illustrates the flaviviral components known to be associated with EVs generated by cells infected with *Flaviviridae.* Secondly, EVs shed by virus-infected cells carry signaling molecules that prepare the microenvironment for virus entry.

### 3.1. EVs in the Transmission of Blood-Borne HCV

In a number of studies, it has been shown that EVs play a role in the transmission of HCV. HCV genomes capable of inducing productive infection in hepatocytes were detected in the exosomes isolated from the sera of infected patients and from HCV-infected hepatocytes [44,45,46,51]. In the study by Bukong et al., it was found that exosomal viral RNA was complexed with miRNA-122, Ago2, and HSP90, and these components could also contribute to the efficacy of viral transmission [44]. Sera and liver tissue exosomes were also found to be carriers of defective HCV genomes that, in the presence of co-expressed full-length viral genomes, could increase HCV replication and likely support viral persistence in clinical settings [58]. Of note, a number of studies evidenced that exosome-mediated viral transmission, although more resistant to neutralizing antibodies, was less efficient than infection with authentic viral particles [46,49,51]. In this context, Longatti et al. demonstrated that the concentration of exosomes from HCV-infected cells was not high enough to establish infection and could only occur through cell–cell contact. At the same time, only 0.1% of the exosomes isolated from the culture media of these cells contained HCV RNA [46]. This aspect points to the need for further studies to establish the role of HCV-containing EVs in the transmission of this virus.

### 3.2. EVs in the Transmission of Arthropod-Borne Flaviviruses

With the exception of HCV, most of the *Flaviviridae* infecting humans are transmitted by arthropods. Given that arboviruses have a complex life cycle with at least two hosts, an alternative route of transmission by EVs would increase their chance of infection and favor their adaptability. Indeed, mosquito and tick cells infected with DENV, ZIKV, and LGTV release EVs that have infectious potential, particularly in human cells, as shown on several cellular models [36,37,39,40,41]. Such EVs can carry viral components, including viral RNA and viral proteins E and NS1 [38]. For example, the saliva of DENV2-infected mosquitoes was shown to contain EVs carrying subgenomic non-coding flaviviral RNA [53]. Its levels were correlated with increased saliva infectivity for human hepatoma cells and dermal fibroblasts [53]. Notably, mosquito transmission of DENV to humans begins with the infection of skin resident cells at the bite site. Since dermal fibroblast cultures mimic these skin resident cells at the bite site, this study demonstrated that EVs from mosquito saliva can enhance viral transmission by preparing the terrain for efficient DENV infection of human cells. Next, EVs from DENV-infected mosquito cells contained not only infectious RNA but also E protein [40]. Interestingly, in the study by Yang et al., DENV infection of insect cells C6/36 increased the levels of C189 tetraspanin, the transmembrane protein involved in exosome formation, and cell-to-cell transmission of DENV was more efficient within C189-containing vacuoles [40]. The role of the other mosquito tetraspanins, CD9 and Tsp29Fb, in EVs-mediated DENV transmission from insect to mammalian cells has also been demonstrated [36,39]. ZIKV-infected C6/36 cells also released EVs containing viral RNA and E protein, which were able to infect both naïve mosquito and mammalian cells, including cultures of monocytes and endothelial microvascular cells [41]. Interestingly, ZIKV E glycoprotein was observed both inside and outside exosomes [41]. As with DENV, tetraspanins, particularly CD63, have been implicated in ZIKV transmission [42].

The member of the tick-borne flaviviral group LGTV has been shown to exit infected tick cells mainly via exosomes containing LGTV RNA and E and NS1 proteins, which were able to transmigrate and infect naïve human skin keratinocytes, the first target of tick bites [37]. Apparently, naïve cells take up infectious exosomes by receptor-mediated endocytosis, as exosome-mediated viral transmission is clathrin-dependent [37]. Interestingly, a heterogenic population of exosomes from tick saliva and salivary glands can inhibit wound healing in human keratinocytes in vivo through the downregulation of CXCL12 and the upregulation of IL-8. This impairs skin barrier functions (cell migration, wound healing, and repair process), ultimately inhibiting the immune response at these sites [59].

### 3.3. EVs in Flaviviral CNS Invasion

The *Flaviviridae* family includes several neurotrophic viruses, ZIKV, WNV, TBEV and JEV, having the ability to infect brain cells [60,61,62,63,64], and DENV, which is supposed to be non-neurotropic but has been shown to enter CNS by hematogenous and axonal routes [65,66,67]. All these flaviviruses are able to cross the blood–brain barrier (BBB) and further invade CNS by means of cell-to-cell transport. EVs are known to cross the BBB [68,69] and also be secreted by CNS cells [70], so they may be involved in the intracellular communication between these cells and thus assist CNS invasion by neurotrophic flaviviruses. Indeed, EVs derived from ZIKV-infected cells were able to disrupt the structure of human brain microvascular endothelial (hcMEC/D3) cell junctions, possibly by inducing the reorganization of VE-cadherin, and probably facilitating ZIKV transmission across the BBB [71]. Next, it has been demonstrated that the EVs of ZIKV-infected primary cortical neurons contain infectious viral genome and E protein and can infect murine cortical neuron cultures [72]. Furthermore, neutral sphingomyelinase SMPD3 regulated the production and release of these EVs, and its inhibition by silencing smpd3 or via treatment with the inhibitor GW4869 reduced ZIKV loads in cortical neurons and neuron-derived exosomes [72]. Neurotrophic WNV and LGTV have been shown to infect murine brain microvascular endothelial cells and neuroblastoma cells and stimulate the secretion of exosomes carrying viral RNA and proteins that can transmit infection to neuronal cells [37]. Thus, EVs may assist *Flaviviridae* in crossing the BBB and their transmission within CNS.

### 3.4. EVs in ZIKV Crossing the Transplacental Barrier

Autophagy, a process associated with viral infection [73], may be regulated by EVs and their contents [74,75]. The autophagy machinery has been shown to function in secretory pathways, including EVs biogenesis [76,77], so autophagy may favor viral transmission. ZIKV infection alters CD63 expression levels and may utilize CD63 in the autophagic secretory pathways, contributing to the release of infectious EVs [42]. Autophagic vesicles may also possibly help ZIKV to cross the transplacental barrier. Curiously, autophagy is a mechanism that protects the placenta from pathogens [78,79], but some viruses, including ZIKV, can hijack it. On a mouse model, the endosomal route was shown to be important for the mother-to-child transmission of ZIKV, and chloroquine or hydroxychloroquine inhibited autophagy-dependent viral replication, effectively preventing maternal-to-fetal transmission of the virus [80,81]. For HCV, induction of autophagy has also been shown to affect endosomal pathways and may support the exosome-mediated release of viral particles [82], and knockdown of autophagy inhibits exosome-mediated viral transmission [83].

## 4. EVs Favor Immune Evasion by *Flaviviridae*

*Flaviviridae* have developed multiple strategies to evade the host immune response, both innate and adaptive [84,85,86,87,88,89]. Viral components are supposed to play a major role in it [85,90]. However, immune evasion may also be associated with other reasons, such as the EVs induced in flaviviral infections. By using EVs, viruses can evade host factors (pattern recognition receptors (PRR), antibodies, immune cells) or modulate their functions.

### 4.1. EVs Favor the Evasion of Innate Immune Recognition and Neutralizing Antibodies

EVs with infectious viral components cay help viruses escape from immune system effectors and establish chronic infection. EVs released from an HCV-infected hepatocyte cell culture contained a fraction of HCV dsRNA intermediates, which decreased RNA recognition and, thus, reduced the activation of the TLR3 signaling pathway [47]. Blocking vesicles release in HCV-positive cells increased intracellular dsRNA levels and restored TLR3 activation, inhibiting viral replication [47].

By using EVs, viruses can evade another host defense mechanism of virus elimination, i.e., neutralizing antibodies. In the studies mentioned above, exosomes carrying HCV RNA could favor viral transmission to naïve cells. This transmission mode has been shown to be resistant to neutralizing antibodies [44,46,51], which may explain, at least in part, the relative ineffectiveness of these antibodies in blocking HCV infection. An interesting effect was observed in the study by Deng et al. in which hepatoma cells and hepatocytes were demonstrated to produce exosomes carrying HCV glycoprotein E2 on their surface, which could sequester anti-HCV neutralizing antibodies and, thus, promote the neutralization escape of infectious HCV particles [50]. The efficiency of production of these E2-coated exosomes was boosted by the expression of syntenin, an intracellular adaptor protein involved in exosome biogenesis [50].

A similar mechanism was demonstrated in DENV infection, which induced the production of autophagy-associated vesicles which were detected in patients sera. These vesicles contained viral RNA and viral proteins E, NS1, prM/M, host lipid droplets and the autophagy marker protein LC-II [52]. DENV-specific neutralizing antibodies had no neutralizing activity against such vesicles, and these vesicles could successfully initiate a new round of infection in the target cells [52]. This phenomenon may explain the inefficiency of neutralizing antibodies upon DENV infection in vivo. The role of autophagy in DENV transmission was illustrated by experiments in DENV-infected autophagy-deficient cells, where a reduction in DENV vesicles formation was observed [52].

### 4.2. EVs Carry Effector Molecules Targeting the Host Immune System

EVs produced during *Flaviviridae* infections may also carry effector molecules with negative effects on host immunity. Exosomes isolated from the plasma of viremic HCV patients were enriched for a specific set of miRNAs, of which miRNA-122-5p and miRNA-222-3p could be correlates of NK degranulation activity [91]. Notably, direct acting antivirals (DAA) therapy helped decrease the levels of these miRNAs and restore NK cells functions [91]. Exosomes produced by DCs infected with a pathogenic strain of DENV (DENV3-5532) contained miRNAs capable of interfering with the mRNA surveillance pathway that normally helps degrade viral RNAs [92]. The authors of the above-mentioned study showing increased infectivity of mosquito saliva with EVs containing DENV non-coding RNAs also proposed that such an effect was due to the inhibition of the IFN type I and III signaling mediated by these EVs [53].

EVs from HCV-infected cells have been shown to inhibit the adaptive immune response. First, HCV-infected hepatocytes were shown to produce TGF-β containing exosomes, which may play a pivotal role in the accumulation of Tfr (T follicular regulatory) cells [93]. This potentially inhibited protective Tfh (T follicular helper) cells’ responses in HCV-infected patients, leading to the suppression of the generation of high-affinity antibody-producing B cells and contributing to viral persistence [93]. Second, exosomes produced by HCV-infected cells were involved in stimulating monocytes to secrete lectin galectin-9, which was elevated in the liver and sera of HCV-patients, inducing the apoptosis of HCV-specific T cells and increasing the levels of inhibitory regulatory T cells [94].

## 5. EV Roles in the Inflammatory Pathogenesis of *Flaviviridae*

*Flaviviridae* pathogenesis is closely associated with inflammation that begins in the infected cells and develops with the subsequent involvement of immune cells. All the types of immune cells involved in inflammatory processes, i.e., macrophages [95,96,97], mast cells [98], DCs [13], neutrophils [99], and T cells [100], have been shown to secrete EVs in response to immune stimuli. Inflammation can modulate the levels of EV secretion and their content, providing them with proinflammatory cytokines and miRNAs and heat shock proteins [101]. In addition, EVs may stimulate bystander cells, further enhancing inflammatory response [101].

Viral tropism determines the tissue specificity of the sites of inflammation development. Flaviviral infections can stimulate EVs that may contribute to tissue-specific inflammatory mechanisms specific to *Flaviviridae*, in particular, liver inflammation, neuroinflammation, and endothelial dysfunction, which will be discussed below. In addition, examples of the anti-inflammatory roles of EVs can be found in Section 5.4, which describes the relationships between EVs and inflammasomes.

### 5.1. EVs in Liver Inflammation

HCV infection becomes chronic in 80–85% of cases and is accompanied by chronic inflammation [102]. In the early stages of liver injury, inflammation plays a role in tissue repair [103], but, over time, excessive inflammation leads to liver cell damage and cell death, with subsequent liver failure, liver fibrosis, regulated by the activation of hepatic stellate cells (HSCs), and hepatocellular carcinoma [104]. EVs can strongly influence these processes and promote the transition from one disease stage to the other through specific immunostimulatory EV cargo [105]. Not only cell-free HCV but also HCV-containing exosomes, isolated from patient sera, were shown to activate the TLR7/8 signaling pathways, so as to transfer HCV particles towards naïve hepatocytes, trigger monocytes differentiation towards macrophages producing mixed M1/M2 cytokines and having M2 surface markers, and, finally, promote the generation of circulating fibrocytes [106], thereby inducing liver fibrosis. Studies in transgenic mice have shown that M2 macrophages promote chronic inflammation in the liver during HCV infection by secreting proinflammatory cytokines IL-6 and TNFα [107]. Exosomes can also deliver specific miRNAs from infected hepatocytes to hepatic stellate cells (HSC), where these miRNAs trigger several pathways associated with liver fibrosis, including TGB-β signaling and the TLR7, and B-cell activation pathways [108,109,110].

### 5.2. EVs in Endothelial Disfunction

#### 5.2.1. DENV

Among all *Flaviviridae*, DENV is the most associated with endothelial cell damage. Most cases of DENV infection cause a mild disease, but, in a subset of patients, it progresses to dengue fever (DF) or to dengue hemorrhagic fever/dengue shock syndrome (DHF/DSS). DHF is characterized by increased capillary permeability, thrombocytopenia, altered leukocytes number and functions, altered hemostasis, and liver damage [111,112]. Patients with DHF grades III and IV may develop spontaneous extensive plasma leakage and hemorrhagic fever, resulting in DSS which can be fatal [113]. DENV can activate endothelial cells either directly, by infecting them, or indirectly, by infecting DCs and monocytes/macrophages, which release a set of soluble proinflammatory cytokines including IL-1β, TNF-α, IL-6, and also IFN-α and IFN-β [111,112,114]. This triggers immune signaling cascades that promote endothelial cell disfunction, ultimately leading to abnormal microvascular function, capillary injury, thrombocytopenia, and vascular leakage with variable multiorgan involvement, neovascularization, and subsequent macrophage recruitment, inflammation, and plaque formation [115,116,117]. During these events platelets may also be activated, further damaging the endothelium and leading to thromboinflammation, characterized by fibrin deposition and thrombus formation [118].

EVs derived from the endothelium, leukocytes, and platelets may have pathological roles in the development of vascular damage. Under abnormal conditions, circulating EVs carrying specific contents may promote endothelial disfunction by increasing the levels of adhesion molecules, reactive oxygen species, and proinflammatory cytokines [119]. This suggests that EVs may be a potential “missing link” in the development of DHF [120,121]. Indeed, in a study, DENV infection activated platelets via highly expressed CLEC2, a tyrosine kinase-coupled C-type lectin [122], which stimulated platelets to release EVs [123]. Interestingly, exosomes further preferentially activated CLEC5A, another C-type lectin, and microvesicles further preferentially activated TLR2 in neutrophils and macrophages [123]. This induced neutrophil extracellular trap (NET) formation and proinflammatory cytokine release, which contributed to increased vascular permeability. The blocking of CLEC5A and TLR2 could inhibit inflammation and lead to the increased survival of DENV-infected mice [123]. Another study showed that both platelets and erythrocytes from DENV-infected patients could shed microparticles and carried viral envelope and NS1 proteins on their surface [54]. Elevated levels of microparticles shed by erythrocytes were directly correlated with DENV severity, particularly during the early acute phase, and could help identify patients with potentially severe disease requiring immediate care [54]. Interestingly, this study demonstrated that a decrease in platelet-derived microparticles was associated with a bleeding tendency [54].

A cytokine storm contributes to the development of severe forms of DENV infection [124], and miRNAs and mRNAs incorporated into exosomes from DENV-infected cells may be among the activating factors in this process [120]. In EVs derived from cells infected with the hemorrhagic strain of DENV, DENV3-5532, the mRNAs related to platelet and endothelial cell activation were enriched as well as the cytokines associated with plasma leakage and DSS, such as IL-6 [92]. The mRNAs of CXCR4, macrophage migration inhibitory factor (MIF), IL-17A, and IL-8, whose increased levels correlated with disease severity, were also found in EVs produced by cells infected with these DENV variants [92]. Macrophages infected with the other dengue strain, DENV-2, emitted exosomes-carrying proteins and miRNAs that induced early changes in the physiology of the endothelium, associated with its alertness status, causing the activation and secretion of proinflammatory mediators, such as TNF-α, IFN-α, IL-6, IL-8, IL-10, IL-12p70, IP-10, and RANTES [56].

#### 5.2.2. ZIKV

Among the other members of the *Flaviviridae*, ZIKV may be associated with certain hemostatic alterations, particularly in the placenta and in the umbilical cord [125,126], or may stimulate endothelial activation associated with BBB breakdown [127]. EVs may contribute to these processes. ZIKV-infected mosquito cells (C6/36 cells) could secrete EVs containing viral RNA and E protein, and these EVs could enter not only naïve insect cells but also human endothelial vascular cells [41]. They activated the coagulation (TF) and inflammation (PAR-1) receptors on their membranes, promoting a proinflammatory and procoagulant cellular state with increased endothelial permeability [41]. These EVs could also promote the differentiation of naïve monocytes, inducing a proinflammatory state with TNF-α expression [41]. Thus, EVs derived from insect cells may contribute to the pathogenesis of ZIKV by promoting inflammation.

#### 5.2.3. NS1 Protein Associated with EVs

Another viral factor that may be detrimental to the endothelial barrier is the NS1 protein of ZIKV and DENV [114,128,129,130], which has been shown to be present in the fraction of EVs emitted by ZIKV- and DENV-infected cells [55]. Furthermore, a study by Safadi et al. showed that NS1 in the form of a dimer can be associated with the surface of excreted exosomes, increasing its availability to the other cells, including endothelial cells [55]. This aspect raises the question of the safety of vaccine platforms based on the exosomes carrying the NS1 protein on the surface.

### 5.3. EVs in Neuroinflammation

Neurotropic *Flaviviridae* ZIKV, WNV, TBEV, and JEV and the non-neurotropic DENV, can cause neuroinflammation, inducing the release of proinflammatory factors and the infiltration of immune cells in the brain [60,61,62,63,64,65]. The exact mechanism of these processes is still unknown, and there are indications that EVs may play a role. In pathological conditions, EVs may modulate neuroinflammatory responses and regulate tissue damage and repair but, at the same time, promote viral replication and disease progression [70]. The ability of *Flaviviridae* to cross the BBB is crucial for neuroinflammation, and we have discussed above that EVs carrying viral components may provide this ability, thereby increasing viral transmission within the CNS.

In addition, there are some other mechanisms that support neuroinflammation. Specifically, JEV-infected microglial cells could secrete EVs carrying the miRNAs let-7a and let-7b, which could be transferred to neurons and stimulate neuronal death through caspase activation [131]. Let-7a/b enhanced the release of TNF-α from microglia through interaction with TLR7, modulating the inflammatory response of microglia, which could also lead to damage in the bystander neuronal cells. The authors of the study attributed these effects to the miRNAs and not to the viral components of the exosomes, as no infectious viral RNA/particles were detected in them [131].

Another mechanism was demonstrated in the model of cells infected with DENV/transfected with DENV NS1 [132]. These cells elicited EVs carrying high levels of miRNA-148, which were internalized by human microglial cells and manipulated the deubiquitinating machinery there, alleviating the inhibition of proinflammatory pathways (TNF-α, NF-kB, IFN-β) and, thus, contributing to neuroinflammation in the CNS [132].

### 5.4. EVs and Inflammasomes

An increasing number of studies have demonstrated that EV secretion correlates with inflammasome activity [133,134,135,136,137,138] and that inflammasome-induced EVs are capable of enhancing inflammatory responses in bystander cells [139]. Inflammasome activation is an integral part of the systemic inflammatory process and accompanies infection by several *Flaviviridae* viruses, including HCV [140,141], DENV [142,143], ZIKV [144,145], WNV [146], and JEV [147]. Viral RNA and proteins are known factors in this activation [141,144,145,148,149,150,151].

The complex of the NLRP3 inflammasome, the most studied inflammasome type, consists of a sensor, an adaptor, and an effector pro-caspase-1 [152,153]. Upon the recognition of priming stimuli, the inflammasome components are expressed, and upon recognition of activating stimuli, they assemble into a functional complex [154,155]. Pro-caspase-1 within the complex then undergoes autoproteolysis to form catalytically active caspase-1, which processes the inactive precursors of IL-1β and IL-18 to their active variants [156,157,158]. Another substrate of caspase-1, gasdermin D, is also processed and further incorporated into the cellular membrane forming the pores in it [159,160]. IL-1β and IL-18 pass through these pores, triggering cascades of inflammatory reactions [161,162]. Pore formation leads to pyroptosis, which is a form of inflammatory cell death [159,160].

Interestingly, pyroptosis also induces a marked release of exosomes [163]. This effect may be due to the caspase-1 dependent cleavage of the trafficking adaptor Rab-interacting lysosomal protein, which promotes the movement of multivesicular bodies towards the cell periphery and induces the selective loading of proinflammatory miRNAs containing an AAUGC motif (hsa-miRNA-124-3p, hsa-miRNA-155-5p, and hsa-miRNA-126-3p) into exosomes [163]. Thus, pyroptosis can induce the release of EVs with proinflammatory roles and enhance the inflammatory response in the organism, but EVs with anti-inflammatory roles are also released. For example, the activation of inflammasomes in macrophages induces the secretion of IFN-β-containing EVs that limit NLRP3 activation in bystander cells, thereby preventing hyperinflammation [138]. In a study by Yan et. al., exosomes derived from umbilical cord mesenchymal stem cells attenuated the production of cleaved caspase-1 in skeletal muscle cells, thereby reducing IL-1β and IL-18 release and pyroptosis [164].

For *Flaviviridae*, to our knowledge, the direct link between inflammasome activation and EV release and cargo has not been demonstrated. Notably, DENV infection can activate NLRP3 inflammasome in platelets [142], and, in the study described above, DENV was shown to activate platelets to emit EVs that have a proinflammatory role on macrophages and neutrophils [165]. It is likely that inflammasome activation during DENV infection could enhance EVs production, which could further enhance inflammatory responses. These points deserve further research not only in relation to DENV infection but also in relation to other *Flaviviridae* infections.

## 6. EVs Help Restrict *Flaviviridae* Infections

In addition to the above mechanisms by which EVs favor viral infections, EVs may also help the host organism to counteract them.

### 6.1. EVs Stimulate Host Innate Immunity

The cargo of EVs produced by immune cells may help to promote the innate immune response. It has been shown that TLR3-activated macrophages can produce exosomes containing members of the miRNA-29 family, which help induce an antiviral state in HCV-infected hepatocytes [166]. Furthermore, EVs isolated from macrophages stimulated with IFN I and II types were able to induce a late long-lasting inhibitory effect on HCV replication, which was also confirmed for EVs from HCV patients under IFN therapy [97]. Exosomes from non-immune cells could also stimulate effector mechanisms against HCV. For example, exosomes secreted by IFN-induced human liver sinusoidal cells [167] and miRNA-containing exosomes secreted by umbilical mesenchymal stem cells had inhibitory effects on HCV replication [168]. Next, HCV RNA delivered by EVs from hepatocytes could induce an innate IFN-α response in neighboring DCs [169]. This is particularly important because these EVs, derived from infected cells, carry immunostimulatory cargo to the professional IFN-producing cells, whose ability to trigger an innate response is not compromised because they are not permissive for infection.

DENV-infected cells were shown to produce exosomes containing interferon-inducible transmembrane protein 3 (IFITM3), which exerted an antiviral activity against DENV in uninfected cells [170]. Human monocyte-derived dendritic cells (mdDC) were found to secrete EVs carrying a set of mRNAs of genes associated with innate immunity, including ATP-dependent helicases (DDX58, DDX60, and DDX60L), chemokines (CXCL10 and CXCL11), and effectors of the type I IFN response (IFI35, IFI44L, IFIT1, IFIT5, IFIT3, and IFITM1) [92]. PBMCs treated with IFN-α were also able to secrete EVs that inhibited the replication of the pathogenic dengue strain DENV3-5532 [92]. WNV could also affect the composition of EVs in infected cells, through both IFN-dependent and IFN-independent pathways, by increasing the EV levels of miRNAs and mRNAs with immunostimulatory and antiviral activities [171].

### 6.2. EVs Attenuate Antibody-Dependent Enhancement

EVs can also play a role in the specific protective mechanisms that help restrict flaviviral infections. EVs derived from human saliva have been shown to be more effective in preventing ZIKV attachment to target cells than EVs derived from other sources [172]. The authors suggest that it protects against ZIKV infection via deep kissing in spite of the susceptibility of oral cells to viral entry and of the high levels of viral RNA and infectious virus present in saliva [172]. Subsequently, ZIKV-infected cells were shown to release EVs carrying viral glycoprotein E on their surface and take up anti-E antibodies, thereby attenuating the antibody-dependent enhancement modulated by these antibodies [57], although not exclusively by anti-ZIKV antibodies but also by cross-reactive antibodies to DENV, as demonstrated in a cell culture and in mouse models [57].

### 6.3. EVs May Favor Antigen Presentation

To our knowledge, there are no studies on *Flaviviridae* investigating EVs in relation to viral antigen presentation, although facilitation of antigen presentation was one of the first functions established for EVs. Professional antigen-presenting cells such as DC and B lymphocytes load antigen peptides onto the MHC class II inside antigen-processing compartments, which are specialized endosomal structures [173]. Their fusion with the plasma membrane releases MHC class II containing intraluminal vesicles as exosomes, can place MHC class II on the plasma membrane where it can be released inside ectosomes [174,175], and, finally, induces antigen-specific MHC class II-restricted T cell responses and modulates the immune response [176,177]. Interestingly, EVs of small and medium sizes could activate mainly CD4+ T cells of a Th1 phenotype and larger EVs-activated Th2 phenotype T cells [178]. Furthermore, EVs containing viral particles captured by neighboring antigen-presenting cells may facilitate the antigen presentation process, even in the absence of complete virions, as has been demonstrated for cancer antigens and some viruses [179,180,181].

## 7. Clinical Application of EVs for the Treatment of *Flaviviridae* Infections

In recent years, the clinical application of EVs as biomarkers and therapeutic carriers has become a developing field of research. Accordingly, the number of clinical trials using EVs has increased, targeting diseases as diverse as cancer, neurodegeneration, inflammation, and immunology. The accumulated knowledge on the EV role in *Flaviviridae* pathogenesis may promote their application in the diagnosis and treatment of infections caused by these viruses.

### 7.1. EVs in Diagnostics and in the Therapy of Flaviviridae Infections

One of the potential clinical applications of EVs useful in the diagnosis and choice of therapy for *Flaviviridae* infections is the use of EV cargo as a biomarker of the disease stage or disease severity. Exosomal miRNAs during HCV infection are an early non-invasive diagnostic biomarker for HCV-related hepatocellular carcinoma [91,182,183,184]. Another example of EV-derived biomarkers are EVs containing specific miRNAs species (let-7e, mir-1261, mir-371b, and mir-4327), which were detected when mdDCs were infected with the hemorrhagic DENV3-5532 strain, but not with the DENV3-290 strain that causes mild dengue [54,92,185]. A ZIKV study using the rhesus macaque trophoblast stem cell model also revealed EV-derived diagnostic non-invasive miRNA markers that may help to identify placental infection [186].

EVs may also be the markers to evaluate the efficacy of antiviral therapy. For example, the level of exosomal negative-sense HCV RNA was indicative of a replication-competent virus and reflected resistance to treatment with IFN/ribavirin, as it was found in the exosomes of treatment non-responders and some treatment-naïve individuals [44]. Exosomal HCV RNA expression also correlated significantly with HCV RNA expression in the sera of HCV-infected patients [48], which may help to predict efficacy of treatment. Exosomal miRNAs-122/155 expression levels may be associated with HCV replication, and the higher the miRNA-122/199a expression, the more positive the therapeutic effect could be expected [48,187]. Furthermore, treatment with DAAs may decrease the levels of some exosomal miRNAs, indicating immune restoration [91]. Finally, miRNA levels may help predict the therapeutic efficacy of DAAs in patients infected with different genotypes of HCV [187].

### 7.2. EVs as Delivery Platforms

EVs are also widely used as platforms for the delivery of bioactive compounds. Their immunogenicity is weaker than that of liposomes or viral vectors, and they are able to cross major physiological barriers including the BBB, making them an attractive basis for the development of therapeutic agents. Here are a few examples of these applications.

Adipose tissue-derived mesenchymal stem cells, which have a high capacity to produce large amounts of exosomes, were transfected with miRNAs and, as a result, secreted exosomes carrying these miRNAs. The miRNAs could be effectively delivered to HCC cells, where they exerted their therapeutic potential, as previously shown in other studies, altering the expression of target genes and making HCC cells more sensitive to chemotherapy [188]. Exosomes secreted by umbilical cord mesenchymal stem cells were previously shown to inhibit HCV replication, probably due to the expression of functional miRNAs including let-7f, miRNA-145, miRNA-199a, and miRNA-221. Thus, these exosomes have been used as adjuvants in combination with IFN-α or telaprevir to enhance their therapeutic effect [168]. The IFITM3-containing EVs described above as potential anti-DENV agents [170] were developed for the treatment of ZIKV and suppressed viremia by a 2-log reduction in pregnant mice [189]. They could also be effectively delivered across the placenta and were shown to suppress ZIKV in the fetus [189].

EVs have also been explored as a platform for vaccination, and some of these successful developments are undergoing clinical trials [190,191]. An antigen can be incorporated into EVs or on their surface, and this is the major challenge in EV-based vaccine development. Two approaches are used for this: the first one is to produce and purify EVs from antigen-expressing cells, and the second is to add an EV-targeting signal to the antigen [192]. Exosome-based vaccine platforms include an intramuscularly delivered DNA vector expressing viral antigen fused to the C-terminus of a mutant variant of the HIV-1 Nef protein, known as the exosome-anchoring protein, which ensures high levels of uptake into the exosomes [193]. This platform has been developed for several viral antigens, including the NS3 of WNV and HCV [193]. In a study, it allowed the antigens to be loaded into the exosomes and, when injected into mice, elicited a highly detectable antigen-specific CD8+ T cell response with cytotoxic activity [193].

### 7.3. EVs as a Target for Inhibitors

EVs and, in particular, their biogenesis can be targeted by chemical inhibitors that help block the pathogenic functions of EVs. For example, blocking tetraspanins, which are essential for exosome formation and the transmission of several *Flaviviridae* including DENV, ZIKV, WNV, and HCV, may be a therapeutic c option to inhibit viral replication [194].

## 8. Conclusions and Perspectives

**Figure 1:**
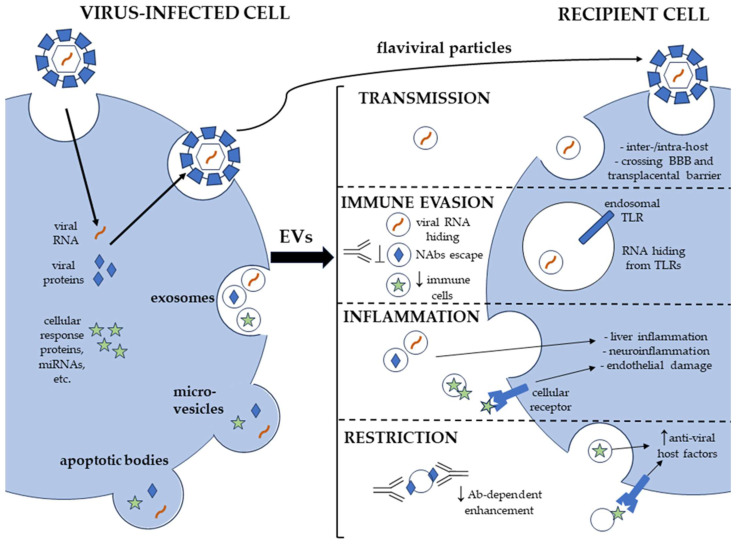
EV roles in *Flaviviridae* pathogenesis.

In this review, we aimed to analyze how EVs may play a role in *Flaviviridae* pathogenesis, which is summarized in the outline of Figure 1.

First, EVs containing viral components facilitate the transmission of *Flaviviridae*, both inter-host and between different cell types within a host. Of note, EVs may help cross the BBB and transmit within the CNS, which is important for neurotropic viruses, and autophagic vesicles generated during ZIKV infection may help cross the transplacental barrier. Second, EVs promote immune evasion either by hiding viral components from innate immune system receptors and neutralizing antibodies or by carrying other molecules that inhibit host immunity. Third, EVs may enhance the proinflammatory responses associated with *Flaviviridae*, contributing to the development of liver inflammation, endothelial damage, and neuroinflammation. At the same time, EVs can also restrict *Flaviviridae* infections by carrying antiviral and immunostimulatory molecules. The overlap of these opposing EV functions should be of particular interest for further investigation. There are also blind spots in topics such as the correlation of EVs’ secretion with inflammasome activation and the role of EVs in the antigen presentation of flaviviral antigens, which should also prompt further studies.

Some common problems associated with EV studies should be considered in further research. These are the methodological drawbacks of EV isolation, including the difficulty in separating EVs from viral particles which share common biophysical properties and from other components. Second, the final fate of secreted EVs cannot always be defined, as, after the secretion and internalization of EVs, their contents may be destroyed inside lysosomes without achieving their objectives. Last but not least, the use of EVs as therapeutic tools raises other puzzling questions, such as the technical challenges of loading cargo inside EVs, quality control, and possible side effects of bioengineered EVs. All these aspects should be considered in future studies.

## Figures and Tables

**Table 1:** The components of *Flaviviridae* viruses associated with EVs.

Virus	Components	Links
HCV	Viral RNA	[44,45,46,47,48]
	E protein (inside)	[45]
	E protein (surface)	[49,50]
	Viral particles	[45,51]
DENV	Viral RNA	[36,52,53]
	E protein (inside)	[36,40,52]
	E protein (surface)	[54]
	prM/M protein (inside)	[52]
	NS1 protein (inside)	[52]
	NS1 protein (surface)	[54,55]
	NS3 protein (inside)	[56]
ZIKV	Viral RNA	[41,42]
	E protein (inside)	[41]
	E protein (surface)	[41,57]
	NS1 protein (surface)	[55]
WNV	Viral RNA	[37]
Langat virus (LGTV)	Viral RNA	[37]
	E protein (inside)	[37]
	NS1 protein (inside)	[37]
